# Large-Scale Profiling of Saponins in Different Ecotypes of *Medicago truncatula*

**DOI:** 10.3389/fpls.2019.00850

**Published:** 2019-07-03

**Authors:** Zhentian Lei, Bonnie S. Watson, David Huhman, Dong Sik Yang, Lloyd W. Sumner

**Affiliations:** ^1^University of Missouri Metabolomics Center, Columbia, MO, United States; ^2^Department of Biochemistry, University of Missouri, Columbia, MO, United States; ^3^Noble Research Institute, Ardmore, OK, United States; ^4^Biomaterials Laboratory, Material Research Center, Samsung Advanced Institute of Technology, Gyeonggi-do, South Korea

**Keywords:** *Medicago truncatula*, ecotypes, triterpene saponin, metabolomics, LC-MS/MS

## Abstract

A total of 1,622 samples representing 201 *Medicago truncatula* ecotypes were analyzed using ultrahigh pressure liquid chromatography coupled to mass spectrometry (UHPLC-MS) to ascertain saponin profiles in different *M. truncatula* ecotypes and to provide data for a genome-wide association study and subsequent line selection for saponin biosynthesis. These ecotypes originated from 14 different Mediterranean countries, i.e., Algeria, Cyprus, France, Greece, Israel, Italy, Jordan, Libya, Morocco, Portugal, Spain, Syria, Tunisia, and Turkey. The results revealed significant differences in the saponin content among the ecotypes. European ecotypes generally contained higher saponin content than African ecotypes (*p* < 0.0001). This suggests that *M. truncatula* ecotypes modulate their secondary metabolism to adapt to their environments. Significant differences in saponin accumulation were also observed between the aerial and the root tissues of the same ecotypes (*p* < 0.0001). While some saponins were found to be present in both the aerial and root tissues, zanhic acid glycosides were found predominantly in the aerial tissues. Bayogenin and hederagenin glycosides were found mostly in roots. The differential spatially resolved accumulation of saponins suggests that saponins in the aerial and root tissues play different roles in plant fitness. Aerial saponins such as zanhic glycosides may act as animal feeding deterrent and root saponins may protect against soil microbes.

## Introduction

Legumes are economically important and widely cultivated crops. They contain relatively high protein content and are important sources of protein for both humans and animals. Their high protein content may be attributed to their unique symbiotic relationship with nitrogen-fixing bacteria. Legumes also produce a vast array of natural products including flavonoids, isoflavonoids, anthocyanins, condensed tannins, lignin, and saponins (Dixon and Sumner, 2003). These natural products play important roles in many important biological processes and are important to legume quality. For example, flavonoids serve as signaling compounds in the symbiotic plant-microbe interactions and induce the expressions of Nod genes in the nitrogen-fixing bacteria. Condensed tannins can prevent bloat associated with animals grazing on legumes with high protein content such as alfalfa and clover. Saponins are triterpene glycosides composed of tritepenoid aglycones (normally referred to as sapogenins) conjugated with various carbohydrate residues. They have been documented to possess anti-fungal, anti-bacterial and anti-insect properties and contribute to plant development and defense against pathogens (Moses et al., 2014). However, due to their hemolytic activity and membrane permeabilization nature, saponins are considered as anti-nutritional. They have been reported to cause bloat, reduce digestibility of proteins, interfere with uptake of nutrients in the gut, and result in reduced weight gain (Francis et al., 2002). These undesired anti-nutritional effects of saponins negatively affect the efficient use of high-protein containing legume forages such as alfalfa and clover as animal feeds. Manipulation of the saponin contents in legumes through genetic engineering and/or molecular breeding may provide an efficient way to improve the nutritional values or field performance of legume forages. However, this effort is hindered by our limited understanding of triterpene saponin biosynthesis. In addition, the effects of growth conditions and developmental stages on levels of individual saponins in legumes are still not clear. Information about saponin variation among the many different ecotypes is also lacking. This knowledge is particularly useful in breeding of low saponin containing legume forages.

LC-MS based metabolomics is ideally suited for the analysis of saponins in complex plant extracts. It has been successfully used in the analyses of saponins in many legumes including *Medicago truncatula* (Huhman et al., 2005; Kapusta et al., 2005a,b), *M. arborea* (Tava et al., 2005), alfalfa (Sen et al., 1998; Bialy et al., 1999), clover (Perez et al., 2013), and soybean. Using LC-Fourier transform ion cyclotron resonance mass spectrometry (FT-ICR MS), Pollier et al. (2011) revealed a complex mixture of saponins in the hairy roots of *M. truncatula*. More recently, saponins in 12 annual Medicago species have been profiled and compared (Tava and Pecetti, 2012). The content of saponins were found to range from 0.38 to 1.35% (dry weight), depending on the species. In addition, differences in the aglycone moieties were observed among the 12 Medicago species. While some aglycones such as bayogenin and hederagenin were found in all the species, some including medicagenic acid and zanhic acid were species-dependent (Tava and Pecetti, 2012). The large number of MS/MS and NMR data collectively generated over the past years by a number of different groups constitutes an important and valuable resource for saponin annotation in *M. truncatula* (Bialy et al., 1999; Kapusta et al., 2005a,b; Tava et al., 2005; Pollier et al., 2011).

**FIGURE 1:**
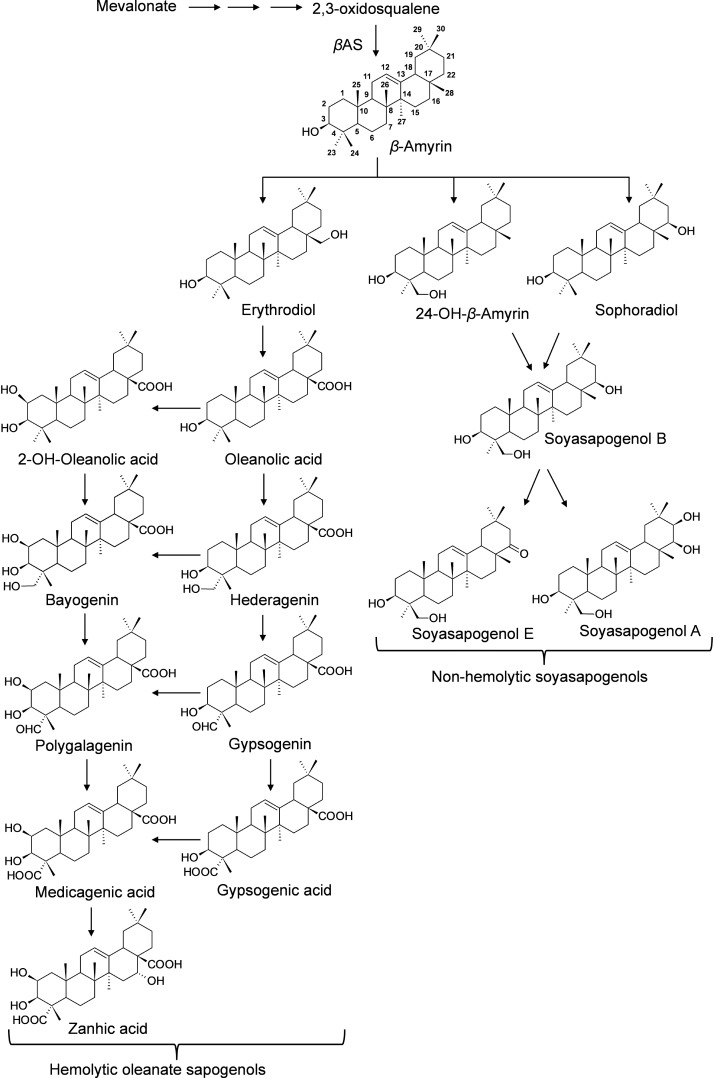
Proposed Biosynthesis of Triterpene Sapogenins in *M. truncatula*. All the sapongenins are derived from beta-amyrin through a series of oxidations that include hydroxylation and subsequent oxidations catalyzed by P450s. Glycosylation of the sapogenins results in the diversity of saponins. In *M. truncatula*, CYP716A12 has been identified to oxidize beta-amyrin to erythrodiol and subsequently to oleanolic acid, and CYP72A68 was found to convert to oleanolic acid, gypsogenin, and subsequently to gypsogenic acid (Fukushima et al., 2013; Tzin et al., 2019). CYP93E2 oxidize beta-amyrin to 24-hydroxy-beta amyrin which is converted to soyasapogenol B by CYP72A61v2 (Fukushima et al., 2013). CYP72A67 responsible for hydroxylation at the C-2 position of oleanolic acid for downstream sapogenin biosynthesis was also identified (Biazzi et al., 2015).

Saponins are broadly classified into hemolytic (oleanates) and non-hemolytic (soyasapogenol) (Figure 1). It is generally believed that the hemolytic activity of olenate saponins is conferred by the presence of a C28-carboxylic group (Voutquenne et al., 2002). Glycosylation of the C28-carboxylic group dramatically reduces and even eliminates the hemolytic activity. Our understanding of triterpene saponin biosynthesis in *M. truncatula* is still very limited. All the saponins are believed to derive from beta-amyrin that is formed through the cyclization of 2,3-oxidosqualene catalyzed by beta-amyrin synthase (Figure 1) (Tava et al., 2011). Hydroxylation and subsequent oxidation of beta-amyrin lead to multiple pentacyclic triterpene aglycones (or sapogenins), glycosylation of which results in diverse and complex saponins. The hydroxylation and oxidation of aglycones are believed to be catalyzed by cytochrome P450 proteins (Tava et al., 2011). In *M. truncatula*, a well-established model legume and close relative to alfalfa (*Medicago sativa*), CYP716A12 has been identified to oxidize beta-amyrin to erythrodiol and then subsequently to oleanolic acid (Carelli et al., 2011; Fukushima et al., 2013). Mutants in CYP716A12, *lha* (*lacking hemolytic activity*), were found to lack hemolytic saponins and only produce non-hemolytic soyasaponins (Carelli et al., 2011). Oxidation of oleanolic acid to hederagenin, gypsogenin, and gypsogenic acid was found to be catalyzed by CYP72A68 (Tzin et al., 2019). Formation of non-hemolytic triterpene aglycones 24-hydroxy-beta-amyrin and soyasapogenol B from beta-amyrin is catalyzed subsequently by CYP93E2 and CYP72A61v2 (Fukushima et al., 2013). A cytochrome P450 (CYP72A67) involved in hemolytic sapogenin biosynthesis was also identified. It was found to be responsible for hydroxylation at the C-2 position of oleanolic acid for downstream sapogenin biosynthesis (Biazzi et al., 2015). However, enzymes involved in the formation of other sapogenins are still unknown. In addition, the effects of the environment on the production of saponins in *M. truncatula* is not clear. To increase our understanding of saponin accumulation in different ecotypes and provide a basis for selecting appropriate *M. truncatula* lines for future correlated gene expression analyses and for the discovery of genes involved in triterpene aglycone biosynthesis, we profiled saponins in 1,622 samples representing 201 *M. truncatula* ecotypes from 14 different countries using UHPLC-MS (Table 1). Differential distributions of saponins in roots and aerial tissues were observed. Zanhic acid saponins were found predominantly in leaves, whereas hederagenin and bayogenin saponins were mostly found in roots. The differential spatial accumulation suggests that different classes of saponins may play different roles in plant defense responses. In addition, different ecotypes were found to accumulate different amounts of saponins, with highest saponin containing ecotypes found mostly in Europe and lowest saponin containing ecotypes were from Africa.

**Table 1:** Geographical origin of the 201 *M. truncatula* inbred lines used for saponin profiling.

Geographic origin	Number of lines
**North Africa**	
Algeria	41
Libya	8
Morocco	10
Tunisia	14
**Europe**	
France	28(13)^a^
Greece	16(2)^b^
Italy	9
Portugal	11(1)^c^
Spain	32
**Middle East**	
Cyprus	9
Israel	5
Jordan	2
Syria	2
Turkey	3
Others of unknown origin	11

*^a^13 out of the 28 lines were from Corsica. ^b^2 out of the 16 lines were from Crete. ^c^1 out of the 11 lines was from Madeira.*

## Materials and Methods

### Biological Materials

Seeds were scarified with sulfuric acid, sterilized with 5% bleach and germinated on damp sterile filter paper. Three days after germination seedlings were transplanted into root cones (Stuewe & Sons Inc.) filled with Turface (BWI Texarkana) which had been rinsed in distilled water and autoclaved. The ecotypes were grown in a growth room under controlled conditions. Day length was 16 h, with a gradual increase in light. The light source included both fluorescent and incandescent bulbs, and light intensity averaged ∼225 μJ. Temperature was set at a constant 21°C. Plants were watered with Broughton and Dilworth (100 ppm N) fertilizer every day. Enough seedlings were planted to analyze four replicates of each ecotype. In most cases, this was accomplished but a few ecotypes grew poorly and three or fewer replicates were harvested. The large number of plants necessitated growth in three separate groups, and A17 and R108 were grown with each group as controls. Plantings were staggered so that all plants could be harvested during the same morning time frame (3–5 h after full light) to insure a uniform position in the diurnal photosynthetic cycle. Plants were harvested at 5 weeks of age before the onset of flowering. Roots and aerial tissues were separated and immediately frozen in liquid nitrogen.

The tissues were lyophilized and dry weights were recorded. All tissues were ground to a fine powder and 10 mg each was weighed for extraction. If plant material was limited, extraction volumes were reduced proportionally. Tissues were incubated with 80:20 methanol:water solution containing 18 μg/ml umbelliferone.

### UHPLC-QTOF-MS

Roots and aerial tissues were lyophilized until dry and ground to a fine powder. Ten milligrams (10 ± 0.06 mg) of powder for all tissues were accurately weighed and extracted on an orbital shaker for 2 h with 1 mL of 80% of methanol containing 18 μg/mL umbelliferone as an internal standard. Samples were centrifuged at 2,900 ×*g* for 30 min at 4°C, and the supernatants were collected. Five microliters of the supernatant were injected into a Waters UPLC system coupled to a quadrupole-time of flight mass spectrometer (QTOF-MS, Waters QTOF Premier). Chromatographic separations were performed on a Waters reverse phase column (2.1 × 150 mm, BEH C18, 1.7 μm particles) using the following gradient: mobile phase B (acetonitrile) increased from 5 to 70% over 30 min, then to 95% in 3 min, held at 95% for 3 min, and returned to 95% mobile phase A (0.05% formic acid in water) for equilibration for 3 min. The flow rate of the mobile phases was 0.56 mL/min, and the column and autosampler temperatures were maintained at 60 and 4°C, respectively. Mass spectral data were acquired from *m/z* 50 to 2,000 in the negative electrospray ionization mode, with the nebulization gas set at 850 L/h (350°C) and the cone gas at 50 L/h (120°C). Raffinose (m/z 503.1612) was used as the reference compound in the independent lock-mass mode, with the lock mass scan (1 s) collected every 10 s for accurate mass measurements. The concentration of raffinose was 50 fmol/mL, and the flow rate 0.2 mL/h.

The raw data files obtained from UHPLC-QTOF-MS analyses were processed with MarkerLynx software (version 4.1, Waters) for mass features extraction and alignment with the following parameters: minimum peak intensity: 500 counts, mass tolerance: 0.05 Da, and retention time window 0.2 min. The peak areas were normalized by dividing each peak area by the value of the internal standard peak (area of metabolite/area of area of internal standard × 1,000). Annotations of metabolites were performed by matching their *m/z* to those of the previously observed saponins in *M. truncatula* (Huhman et al., 2005; Kapusta et al., 2005a,b; Pollier et al., 2011). Retention time was also used in the Rt-m/z pair matching when it was available. Tandem MS was performed on a number of saponins, mainly the most abundant ones, to validate the annotations by matching to previously reported MS/MS data. The MS/MS experiments were performed using a UHPLC-Bruker QTOF MS. The tandem data were compared to previously published data for identification confirmation (Kapusta et al., 2005a; Pollier et al., 2011). The normalized data (i.e., data normalized to the internal standard) were used for statistical analyses. Multivariate statistical analyses were performed using JMP software from SAS (Cary, NC). Tukey HSD (Tukey Honest Significant Differences) was performed using TukeyHSD function in *r*, and the not significantly different groups were labeled with the same letters using the HSD.test function in the “agricolae” package.

## Results and Discussion

**FIGURE 2:**
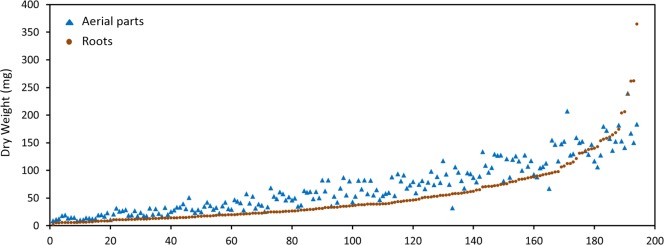
Dry weight distribution of the *M. truncatula* ecotypes. A great majority of the ecotypes (92.3%) were found to produce more aerial tissues (closed triangles) than roots (closed squares) by weight. Significant difference in the dry weight was observed among the ecotypes (*p* < 0.0001) with the highest total dry weight being 548.98 ± 68.25 mg (HM174, Spain) and the lowest 6.42 ± 9.43 mg (HM095, France). Significant positive correlation between the aerial and the root dry weight was also observed (*r* = 0.86, *p* < 0.0001), indicating that ecotypes that produced more aerial tissues also tended to produce more root tissues.

The *M. truncatula* ecotypes (201 lines, 1,622 plants) analyzed in this work represented 14 geographical origins, i.e., Algeria, Cyprus, France, Greece, Israel, Italy, Jordan, Libya, Morocco, Portugal, Spain, Syria, Tunisia, and Turkey (Table 1). The raw data are freely available for download at https://sumnerlab.missouri.edu/download/. Aerial and root tissues were separated, lyophilized and weighed. The dry weight for each ecotype’s aerial and root tissues is shown in Figure 2. Almost all the ecotypes (93.3%) were found to produce more aerial tissues than roots by weight. Significant difference in the average total dry weight was observed among the ecotypes (*p* < 0.0001), with a 90 fold difference between the lowest line (HM095, France, 6.42 ± 9.43 mg, mean ± standard deviation, *n* = 5) and the highest line (HM174, Spain, 548.98 ± 68.25 mg, mean ± standard deviation, *n* = 5). It was also found that there was a significant positive correlation between the aerial and the root dry weight (*r* = 0.86, *p* < 0.0001), indicating that ecotypes producing more aerial tissues also tended to produce more root tissues. The average total dry weight for ecotypes of the same country of origin is shown in Table 2. Most of the North African and Middle East ecotypes appeared to produce less biomass compared to the European ecotypes, but the difference was not statistically significant (*p* = 0.3). This is due to large variations of dry weight among the ecotypes from the same country as evidenced by the large standard deviation associated with each average dry weight (Table 2). Thus, the dry weight of ecotypes, when grown in greenhouse, appeared to be ecotype specific and did not show a statistically significant correlation between their dry weight and their geographic origin. This is probably due to the different growth rates of the different ecotypes. For example, the two well characterized and mostly used ecotypes, A17 and R108, have previously been found to have different seed-to-seed generation times (Hoffmann et al., 1997). The generation time of R108 was 12–14 weeks, which is about 3 weeks shorter than A17. Ecotypes with even shorter seed generation times (e.g., 7 weeks shorter than A17) was also found (Hoffmann et al., 1997). As the ecotypes were harvested at the same time and not at the same developmental stage, the different generation time among the ecotypes may contribute to the high variance observed (Table 2). Although it was desirable to harvest all the ecotypes at the same developmental stage, it was simply not feasible given the scale of this experiment.

**Table 2:** Average total dry weights and their standard deviations of ecotypes.

Country of origin	Average aerial dry weight (mg/line)	Standard error	Average root dry weight (mg/line)	Standard error
Algeria	61.21	6.45	45.98	7.37
Cyprus	50.52	11.80	22.87	4.81
France	91.93	11.89	81.11	14.31
Greece	77.74	10.83	50.11	8.89
Israel	104.72	36.14	87.20	38.89
Italy	91.79	13.59	62.93	17.87
Jordan	57.66	17.53	18.20	5.73
Libya	69.71	9.74	42.79	6.35
Morocco	92.91	13.07	62.68	9.35
Portugal	81.28	10.80	56.74	16.68
Spain	77.23	9.59	62.67	12.40
Syria	51.49	31.00	25.63	12.68
Tunisia	68.26	13.62	52.48	13.29
Turkey	71.74	10.42	28.82	6.80

*The average total dry weight was calculated by averaging the average weights of all individual M. truncatula lines from the same country of origin.*

**FIGURE 3:**
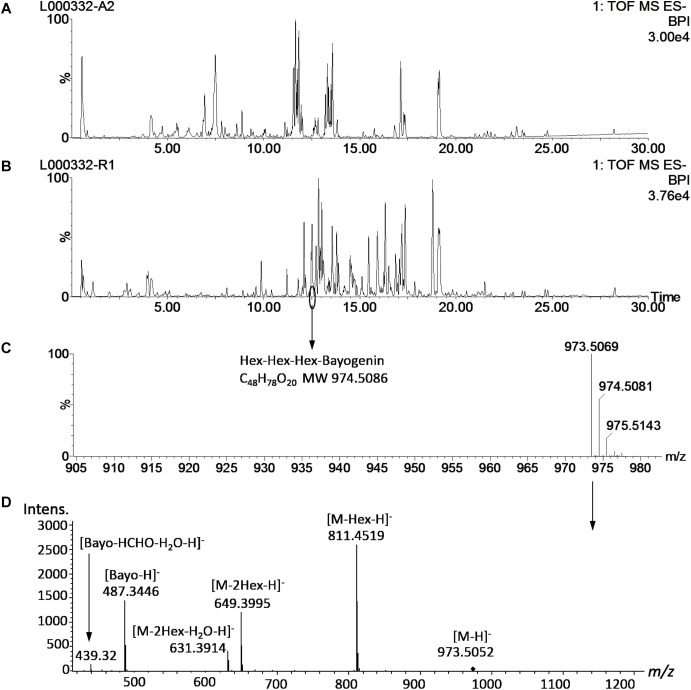
Representative UHPLC-MS metabolite profiles of *M. truncatula.* **(A)** UHPLC-ESI-MS chromatogram of aerial tissue extract of *M. truncatula* HapMap 135 (line number: L000332, country of origin: Israel). **(B)** UHPLC-ESI-MS chromatogram of root tissue extract of HapMap 135. **(C)** Mass spectrum of a peak at Rt 12.48 min in the root tissue extract showing *m/z* at 973.5183 and identified as hexose-hexose-hexose-bayogenin. **(D)** MS/MS spectrum of the precursor ion at *m/z* 973.5 at a collision energy ramped from 55 to 90 V, confirming glucose-glucose-glucose-bayogenin. MS spectrum was acquired with a QTOF Premier (Waters) and MS/MS spectrum with a MaXis Impact QTOF (Bruker Daltonics). The data show that hexose-hexose-hexose-bayogenin is predominantly accumulated in the roots of *M. truncatula*. Bayo: bayogenin; Hex: heoxse.

Saponin profiling of the aerial and root tissues was performed in a random manner and spanned over a period of 5 months (November 2012–March 2013). The data are shown in Supplementary Tables 1,2. Reproducibility is always a concern when large-scale experiments of this magnitude are performed. To monitor the reproducibility, a blank wash and a quality control (QC) mixture were performed every 10 samples to monitor any carry-over or changes in instrumental response. An internal standard solution (extraction solution only) was also analyzed every 20 samples. Eighty-seven injections of the internal standard solution were made during the 5-months of analyses and the responses were used to calculate the relative standard deviation (RSD) to quantify reproducibility. The RSD was determined to be 15.3%, comparable to a previously reported value (15.9%) in a metabolomics project (Kirwan et al., 2013) and below that (20%) recommended for large-scale metabolomics (Food and Drug Administration [FDA], 2001; Zelena et al., 2009). Annotation of saponins was performed by matching the mass features’ *m/z* to those of saponins found in *M. truncatula* and then confirmed by MS/MS (Figure 3). Figure 3 shows a representative UHPLC-MS chromatogram of the aerial and root tissues of HapMap 135 (line number: L000332, country of origin: Israel). Significant differences were observed between the metabolic profiles of the aerial and root tissues (Figure 3A,B). For example, a peak (*m/z* 973.5069_Rt12.48 min) found predominantly in the root tissues was annotated as glucose-glucose-glucose-bayogenin and confirmed by tandem MS (Figure 3C,D). The relative abundances of saponins (area of metabolite peak normalized to that of the internal standard in the sample) were used for statistical analysis such as principal component analysis (PCA) and multiple mean comparisons. The results showed that there was significant difference in the saponin content among different ecotypes (*p* < 0.0001) as well as between the aerial and the root tissues of the same ecotypes (*p* < 0.0001). These data indicated that the geographical origin has an impact on the production of saponin in plants and that saponins differentially accumulated in roots and aerial tissues. Their differences and biological implications are discussed below.

### Saponin Content Among Ecotypes

**FIGURE 4:**
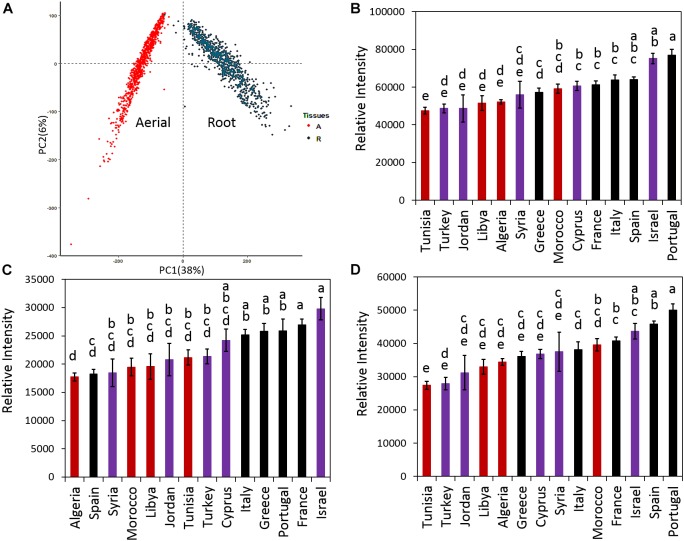
Distribution of saponins in *M. truncatula* ecotypes. **(A)** Principal component analysis (PCA) of detected saponins in all samples. **(B)** Average total saponin content in ecotypes of the same country of origin. **(C)** Average aerial saponin content in ecotypes of the same country of origin. **(D)** Average root saponin content in the ecotypes of the same country of origin. Error bar: standard error. The data show that ecotypes originated from Europe contain more saponins than those originated from North Africa and Middle East. Countries that have the same letters are not significantly different. Red color: African ecotypes. Purple color: Middle East ecotypes. Black color: European ecotypes.

Principal component analysis was performed for all samples and means for the total, aerial and root saponin content in ecotypes of the same country of origin were calculated (Figure 4). Figure 4 shows the PCA results (Figure 4A), mean of total saponins (Figure 4B), mean of aerial saponins (Figure 4C) and mean of root saponins (Figure 4D) in ecotypes from the same country. There was a significant difference in the total saponin content among the ecotypes of different countries (*p* < 0.0001). The lowest saponin-containing ecotypes were found to originate from Tunisia and contained only 60% of the amount of saponins in the highest saponin-containing ecotypes (Portugal). Figure 4B also shows that the low saponin-containing ecotypes were mostly from Africa except those originating from Turkey. These ecotypes (Algeria, Libya, Tunisia, and Turkey) contained comparable amounts of total saponins (*p* = 0.4). This may be explained by the similar environments such as similar climates, seasonal changes and soil conditions in these countries as most of them are in the African continent. In contrast, ecotypes originating from Israel and European countries such as France, Italy, Portugal, and Spain typically contained higher amount of saponins. The two highest saponin-containing ecotypes were from Portugal and Israel. This distinct difference between the African and the European ecotypes indicated a clear geographic segregation of *M. truncatula* around the Mediterranean area in terms of saponin production, with the ecotypes in the north region (Europe) segregated from those in the south region (Africa). Similar segregation has also been observed in a previous microsatellite diversity study of 346 inbred lines of *M. truncatula* ecotypes that revealed a stratification of the *M. truncatula* population between the North and the South of the Mediterranean basin (Ronfort et al., 2006). It was further suggested that the *M. truncatula* colonization of the Mediterranean region was via two routes from its original habitat around the Middle East (Ronfort et al., 2006). However, the molecular mechanism responsible for the difference, i.e., higher saponin contents in European ecotypes and lower saponin contents in the African ecotypes, is not clear. Plant secondary metabolism is complex and influenced by both biotic and abiotic stimuli. Plants under different environments can modulate their secondary metabolism to increase their fitness and adapt to the environments. The effect of environments on plant saponin biosynthesis has been recently reviewed (Szakiel et al., 2011). Both biotic and abiotic factors (e.g., temperature, light, humidity, water availability, soil fertility, insects, herbivores, competition from neighboring plants) and their interactions all can affect saponin biosynthesis. For example, saponin content was found higher in aphid-infested alfalfa compared to the uninfested alfalfa (Goławska et al., 2012). A study of herbivore-induced responses in alfalfa further indicated that saponin content increased with higher herbivore densities (Agrell et al., 2003). Methyl jasmonate treatment that mimics mechanical wounding of plants was also found to increase the production of saponins in *M. truncatula* (Suzuki et al., 2002), suggesting that grazing also results in higher saponin content in forage legumes. The environmental abiotic factors also affect saponin content significantly. It has been shown that saponin content decreased significantly in medicinal plants exposed to drought stress (Solíz-Guerrero et al., 2002; Zhu et al., 2009) and the application of an appropriate amount of inorganic fertilizer was able to partly restore saponin content in *Bupleurum* (Zhu et al., 2009). In *Brachiaria*, the main forage for ruminants cultivated worldwide in both tropical and subtropical climates, the saponin content was found to correlate negatively with the duration of sunshine and maximum ambient temperature, but positively with relative humidity (Lima et al., 2012). This suggests that the combination of high temperature, long duration of sunshine and arid condition in Africa might, at least in part, be responsible for the lower saponin content in the African ecotypes. A recent study using over 20,000 annotated genes from *M. truncatula* showed that genes involved in defense against pathogens and herbivores constituted the single largest functional class of genes under positive selection in adaptive evolution (Paape et al., 2013). Other genes under positive selection included those involved in mediating symbiotic relationship with rhizobia and one-third of the annotated histone-lysine methyltransferases that could be involved in epigenetic modifications (Paape et al., 2013). The relatively higher saponin content in European ecotypes may reflect such a positive selection of genes related to disease and defense response as saponins are anti-feeding and anti-microbial (Da Silva et al., 2012; Goławska et al., 2012). Significant variations in the response of *M. truncatula* ecotypes to *Verticillium albo-atrum*, a soil-borne pathogenic fungus, have been reported recently (Ben et al., 2013). Comparison of the resistant and susceptible *M. truncatula* ecotypes led to the identification of three QTLs associated with resistance to the *Verticillium* wilt in the resistant lines. The resistance appeared to be selected within environments as it did not seem to correlate with the population structure (Ben et al., 2013).

In addition to the total saponin content, the aerial and root tissues of the African ecotypes were also found to contain significantly less saponins than the European ecotypes (*p* < 0.0001) (Figure 4C,D). In the aerial tissues, the lowest saponin containing ecotypes were found to be from Algeria. They contained only 60% of the saponins found in the highest saponin-containing ecotypes (Israel). In roots, Tunisia’s ecotypes contained the least amount of saponin, only 55% of the saponin in the highest saponin-containing ecotypes (Portugal). Figure 4 also reveals that roots contained higher saponin content than the aerial parts. For example, compared to their aerial parts, the roots of Portugal’s ecotypes contained about twice the amount of saponins (Figure 4C,D). The differences between the aerial and the root tissues were not only quantitative but also qualitative as evidenced by the distinct saponin profiles in the aerial parts (Figure 3A) and the roots (Figure 3B). This was further supported by PCA analysis that showed the aerial tissues were clearly segregated from the root tissues (Figure 4A), suggesting that the difference between the aerial and the root tissues would be greater than the difference among ecotypes. The differential accumulation of saponins in the aerial and the root tissues may reflect the different roles of these saponins in plant fitness and defense response as discussed below.

### Differential Accumulation of Saponins in the Aerial and Root Tissues

**FIGURE 5:**
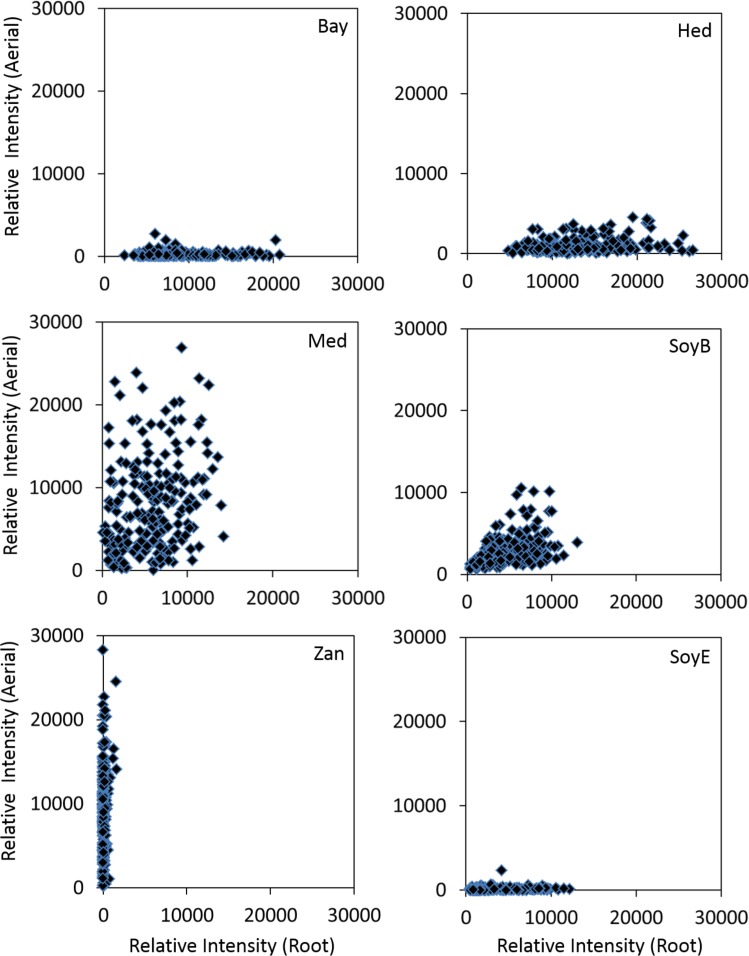
Accumulations of various classes of saponins in the aerial (*Y*-axis) and root (*X*-axis) tissues of different *M. truncatula* ecotypes. Bay, bayogenin glycosides; Hed, hederagenin glycosides; Med, medicagenic acid glycosides; SoyB, soyasaponin B glycosides; Zan, zanhic acid glycosides; SoyE, soyasaponin E glycosides. Zanhic acid glycosides were predominantly found in the aerial tissues while bayogenin and hederagenin glycosides were mostly accumulated in the roots suggesting differential physiological roles for these compounds. In contrast, medicagenic acid glycosides are found in both the aerial and root tissues.

The results of the saponin profiling in the aerial and the root tissues are shown in Figure 5. Figure 5 shows the accumulation of individual classes of saponins (i.e., bayogenin glycosides, hederagenin glycosides, medicagenic acid glycosides, soyasaponin B glycosides, soyasaponin E glycosides, and zanhic acid glycosides) in the aerial (*Y*-axis) and the root (*X*-axis) tissues of the ecotypes. It reveals clear differences in the spatial accumulation of saponins. Regardless of the country of origin, bayogenin glycosides, hederagenin glycosides and soyaspogenin E glycosides were mostly found in the roots, while zanhic acid glycosides were only detected in the aerial tissues. In contrast, medicagenic acid glycosides and soyasaponin B glycosides were found in both the aerial and root tissues although the aerial tissues appeared to contain more medicagenic acid glycosides. The finding is similar to a previous report that zanhic acid glycosides, medicagenic acid glycosides and soyasaponin B glycosides were found to be the three dominant groups of saponins in *M. truncatula* foliar tissues (Kapusta et al., 2005b; Confalonieri et al., 2009). It is also consistent with several previous reports that zanhic acid was the major aglycone in the aerial parts of *M. truncatula* (Kapusta et al., 2005a,b) and zanhic acid glycosides could not be detected in the roots of *M. truncatula* (Confalonieri et al., 2009). A more recent study of *M. truncatula* hairy roots showed that the zanhic acid glycosides were also absent in the hairy roots (Pollier et al., 2011). Zanhic acid glycosides are therefore leaf-specific saponins. This suggests that P450 enzyme(s) involved in the biosynthesis of zanhic acid can best be studied using leaf tissues. Comparisons of P450 gene expression profiles between the aerial and root tissues may facilitate the identification of enzymes converting medicagenic acid into zanhic acid. Zanhic acid formation is the last step of the sapogenol biosynthesis pathway (Figure 1). The accumulation of zanhic acid glycosides and the lack of other sapoinins in the earlier steps of the pathway in the aerial tissues suggest that the physiological role of zanhic acid glycosides cannot be substituted for by other saponins such as hederagenin and bayogenin glycosides. The differential accumulation of saponins in the aerial and the root tissues may reflect the effect of the different environments on the aerial parts and the roots. Compared to roots, one of the unique stresses that the aerial parts face is herbivore feeding and wounding. This suggests that zanhic acid glycosides may be potent anti-feeding metabolites. Indeed, some zanhic acid glycosides have been found to be the most bitter and throat-irritating components among the complex sapoinins in alfalfa (Oleszek et al., 1992) and the most active compounds in disrupting the transmural potential difference in mammalian small intestine (Oleszek et al., 1994). Therefore, zanhic acid glycosides are generally considered as anti-nutritional and the major anti-feeding agents against herbivores in leaves. Similar to alfalfa, medicagenic acid glycosides were also found in the aerial parts of *M. truncatula* ecotypes and their amounts were higher in the aerial parts than in the roots (Figure 5). Unlike zanhic acid glycosides, medicagenic acid glycosides have been reported to possess, in addition to the anti-feeding property, a broad and strong anti-microbial activity (Oleszek et al., 1990; Oleszek, 1996; Avato et al., 2006; Jarecka et al., 2008). For example, medicagenic acid glycosides strongly inhibited the growth of *Trichoderma viride*, a fungus that is highly sensitive to alfalfa saponins and has been traditionally used to quantify saponins (Zimmer et al., 1967). In contrast, zanhic acid glycosides were found inactive against a wide range of fungi including *T. viride*; even at higher concentrations (Oleszek et al., 1992; Oleszek, 1996). Because the difference between medicagenic acid and zanhic acid is the presence of C16-hydroxy group in zanhic acid, it has been suggested that the C16-hydroxy group is responsible for the strong bitter taste but low anti-fungal activity of zanhic acid glycosides (Oleszek, 1996). Soyasaponin B glycosides were also found to accumulate in the aerial parts and the roots (Figure 5). This is consistent with the previous report that soyasaponin B glycosides were found in both aerial and root tissues of alfalfa (Sen et al., 1998). Soyasaponin B glycosides are non-hemolytic saponins. They have been reported to possess anti-feeding and antifungal activities. The combination of saponins of distinct functions in leaves provides an excellent defense against herbivores and fungal attacks. Indeed, incorporation of dried alfalfa leaf tissue in their diet significantly inhibited growth and development of larvae of the European corn borer (*Ostrinia nubilalis*). In contrast, saponin fractions isolated from alfalfa root tissues, when incorporated into their diet at equivalent concentrations, had little effect on larvae development although they inhibited their growth (Nozzolillo et al., 1997). Feeding *Spodoptera littoralis* (Egyptian Cotton Leafworm) larvae with a diet supplemented with saponins isolated from alfalfa also significantly reduced their growth, fecundity and fertility and increased their mortality (Adel et al., 2000). Medicagenic acid and its glycosides were found to be much more effective in inhibiting larvae than hederagenin and its glycosides which are normally accumulated in roots. In a more recent study, the number of aphids infesting alfalfa was found to be inversely related to the contents of zanhic acid and medicagenic acid glycosides in the leaves (Goławska et al., 2012). It was also demonstrated that these compounds were induced upon aphid infestation, indicating their anti-feeding properties. All these suggest that saponins in leaves have been tailored to defend against herbivore feeding through increased bitterness and toxicity.

Compared to leaf saponins, root saponins consisted of a different set of triterpene glycosides, with the major difference being the absence of zanhic acid glycoisdes and the presence of bayogenin glycosides and hederagenin glycosides (Figure 5). This suggests that P450s responsible for converting medicagenic acid to zanhic acid is not active in roots. This is not surprising as leaves and roots are in different environments and may require different saponins to respond to different abiotic and biotic stress. The absence of the bitter zanhic acid glycosides in roots suggests that anti-feeding is less important in roots than in leaves. The abundance of bayogenin and hederagenin glycosides found in roots suggests that these root-specific compounds are important to root fitness. We hypothesize that they likely serve important roles in defense against soil pathogenic microbes. Indeed, saponins from *Medicago hybrida* roots were found to substantially inhibit six pathogenic fungi *Botrytis cinerea*, *Botrytis tulipae*, *Fusarium oxysporum* f. sp. *callistephi*, *F. oxysporum* f. sp. *narcissi*, *Phoma narcissi*, and *F. oxysporum* Schlecht (Saniewska et al., 2006). Similarly, saponins isolated from roots of alfalfa were also found to have strong anti-fungal activity (Jarecka et al., 2008). The strong antifungal activity was attributed to some hederagenin and medicagenic acid glycosides (Saniewska et al., 2006; Jarecka et al., 2008). Bayogenin glycosides have also been reported to possess anti-fungal activity (Martyniuk and Biały, 2008). Their inhibitory effects against *Cephalosporium gramineum*, a soil fungus that infects roots of plants, were markedly higher than their similar hederagenin glycoside counterparts (Martyniuk and Biały, 2008). Bayogenin differs with hederagenin only in that it possesses a hydroxyl group at C2 position (Figure 1). The hydroxyl groups at C2 and C3 positions are important for antifungal activities, possibly due to the increased polarity and solubility (Oleszek, 1996). Selective methylation of the hydroxyl groups in medicagenic acid showed that the hydroxyl group at C3 position is essential for antifungal activity (Levy et al., 1989). While glycosylation of the C3 hydroxyl group did not affect the overall antifungal activity, methylation or acetylation resulted in a significant loss of the antifungal activity. This suggests that the polarity of these compounds is important in their antifungal activity. The spatially differential accumulation of saponins in the aerial parts and roots shows that plant secondary metabolism is flexible and adaptable. Different tissues of the same plant can accumulate different sets of metabolites to increase their fitness and adapt to the environments. In *M. truncatula*, the bitter herbivore deterrent zanhic acid glycosides are predominantly found in the aerial tissues while the anti-fungal agents such as hederagenin and bayogenin glycosides are mostly found in roots to defend against soil borne fungi. In contrast, medicagenic acid glycosides, the broad spectrum and strong anti-microbial compounds, are accumulated in both the aerial and root tissues.

## Conclusion

Saponin profiling of 201 *M. truncatula* ecotypes revealed a clear differential spatial accumulation of saponins in the aerial parts relative to the roots. Zanhic acid glycosides were only found in the aerial tissues. In contrast, bayogenin glycosides, hederagenin glycosides, and soyasaponin B glycosides were predominantly accumulated in roots, thus suggesting interesting ecological roles for these compounds in plant defense. Overall, zanhic acid glycosides, medicagenic acid glycosides and soyasaponin B glycosides were the three major triterpene saponins found in the aerial parts, while medicagenic acid glycosides, bayogenin glycosides, hederagenin glycosides, SoyE glycosides, and SoyB glycosides constituted the major root saponins. Although medicagenic acid glycosides were found in both the aerial and root tissues, aerial parts were found to contain more medicagenic acid glycosides. Significant correlation between the quantity of saponins and the ecotypes’ country of origin was observed. The European ecotypes were found to contain higher content of saponins than most of the African and the Middle-East ecotypes. This dataset also represents an extremely valuable resource for discovery of biosynthetic genes and deciphering saponin biosynthesis. Based on the correlation analysis of the metabolic profiling data and gene expression data, a number of P450 genes have been selected for further characterization to elucidate their roles in saponin biosynthesis in *M. truncatula* (Tzin et al., 2019).

## Data Availability

All processed data for this study are included in the manuscript and/or the Supplementary Files.

## Author Contributions

LS conceptualized and designed the experiments, and secured the funding. BW and DY grew, harvested, dried, and ground the samples. DH extracted the ground samples and collected the LCMS data. ZL processed the data and drafted the manuscript. All authors were involved in the implementation of the experimental design and contributed to the final version of the manuscript.

## Conflict of Interest Statement

The authors declare that the research was conducted in the absence of any commercial or financial relationships that could be construed as a potential conflict of interest.

## References

[B1] Adel M. M. Sehnal F. Jurzysta M. (2000). Effects of alfalfa saponins on the moth *Spodoptera littoralis*. *J. Chem. Ecol.* 26 1065–1078. 15672673

[B2] Agrell J. Oleszek W. Stochmal A. Olsen M. Anderson P. (2003). Herbivore-induced responses in alfalfa (*Medicago sativa*). *J. Chem. Ecol.* 29 303–320. 12737260 10.1023/a:1022625810395

[B3] Avato P. Bucci R. Tava A. Vitali C. Rosato A. Bialy Z. (2006). Antimicrobial activity of saponins from Medicago sp.: structure-activity relationship. *Phytother. Res.* 20 454–457. 16619355 10.1002/ptr.1876

[B4] Ben C. Toueni M. Montanari S. Tardin M. C. Fervel M. Negahi A. (2013). Natural diversity in the model legume *Medicago truncatula* allows identifying distinct genetic mechanisms conferring partial resistance to Verticillium wilt. *J. Exp. Bot.* 64 317–332. 10.1093/jxb/ers337 23213135 PMC3528038

[B5] Bialy Z. Jurzysta M. Oleszek W. Piacente S. Pizza C. (1999). Saponins in alfalfa (*Medicago sativa* L.) root and their structural elucidation. *J. Agric. Food Chem.* 47 3185–3192. 10552628 10.1021/jf9901237

[B6] Biazzi E. Carelli M. Tava A. Abbruscato P. Losini I. Avato P. (2015). CYP72A67 catalyzes a key oxidative step in *Medicago truncatula* hemolytic saponin biosynthesis. *Mol. Plant* 8 1493–1506. 10.1016/j.molp.2015.06.003 26079384

[B7] Carelli M. Biazzi E. Panara F. Tava A. Scaramelli L. Porceddu A. (2011). Medicago truncatula CYP716A12 is a multifunctional oxidase involved in the biosynthesis of hemolytic saponins. *Plant cell* 23 3070–3081. 10.1105/tpc.111.087312 21821776 PMC3180811

[B8] Confalonieri M. Cammareri M. Biazzi E. Pecchia P. Salema Fevereiro M. P. Balestrazzi A. (2009). Enhanced triterpene saponin biosynthesis and root nodulation in transgenic barrel medic (*Medicago truncatula* Gaertn.) expressing a novel β-amyrin synthase (AsOXA1) gene. *Plant Biotechnol. J.* 7 172–182. 10.1111/j.1467-7652.2008.00385.x 19055609

[B9] Da Silva P. Eyraud V. Carre-Pierrat M. Sivignon C. Rahioui I. Royer C. (2012). High toxicity and specificity of the saponin 3-GlcA-28-AraRhaxyl-medicagenate, from *Medicago truncatula* seeds, for *Sitophilus oryzae*. *BMC Chem. Biol.* 12:3. 10.1186/1472-6769-12-3 22536832 PMC3388004

[B10] Dixon R. A. Sumner L. W. (2003). Legume natural products: understanding and manipulating complex pathways for human and animal health. *Plant Physiol.* 131 878–885. 12644640 10.1104/pp.102.017319 PMC1540287

[B11] Food and Drug Administration [FDA] (2001). *FDA Guidance for Industry: Bioanalytical Method Validation*. Silver Spring, MA: FDA.

[B12] Francis G. Kerem Z. Makkar H. P. Becker K. (2002). The biological action of saponins in animal systems: a review. *Br. J. Nutr.* 88 587–605. 12493081 10.1079/BJN2002725

[B13] Fukushima E. O. Seki H. Sawai S. Suzuki M. Ohyama K. Saito K. (2013). Combinatorial biosynthesis of legume natural and rare triterpenoids in engineered yeast. *Plant Cell Physiol.* 54 740–749. 10.1093/pcp/pct015 23378447

[B14] Goławska S. Łukasik I. Wójcicka A. Sytykiewicz H. (2012). Relationship between saponin content in alfalfa and aphid development. *Acta Biol. Crac. Ser. Bot.* 54 39–46. 10.2478/v10182-012-0022-y

[B15] Hoffmann B. Trinh T. H. Leung J. Kondorosi A. Kondorosi E. (1997). A new medicago truncatula line with superior in vitro regeneration, transformation, and symbiotic properties isolated through cell culture selection. *Mol. Plant Microbe Interact.* 10 307–315.

[B16] Huhman D. V. Berhow M. A. Sumner L. W. (2005). Quantification of saponins in aerial and subterranean tissues of *Medicago truncatula*. *J. Agric. Food Chem.* 53 1914–1920. 15769113 10.1021/jf0482663

[B17] Jarecka A. Saniewska A. Biały Z. Jurzysta M. (2008). The effect of *Medicago arabica*, *M. hybrida* and *M. sativa* saponins on the growth and development of *Fusarium oxysporum* schlecht f. sp. tulipae Apt. *Acta Agrobot.* 61:9.

[B18] Kapusta I. Janda B. Stochmal A. Oleszek W. (2005a). Determination of saponins in aerial parts of barrel medic (*Medicago truncatula*) by liquid chromatography-electrospray ionization/mass spectrometry. *J. Agric. Food Chem.* 53 7654–7660. 16190612 10.1021/jf051256x

[B19] Kapusta I. Stochmal A. Perrone A. Piacente S. Pizza C. Oleszek W. (2005b). Triterpene saponins from barrel medic (*Medicago truncatula*) aerial parts. *J. Agric. Food Chem.* 53 2164–2170. 15769151 10.1021/jf048178i

[B20] Kirwan J. A. Broadhurst D. I. Davidson R. L. Viant M. R. (2013). Characterising and correcting batch variation in an automated direct infusion mass spectrometry (DIMS) metabolomics workflow. *Anal. Bioanal. Chem.* 405 5147–5157. 10.1007/s00216-013-6856-7 23455646

[B21] Levy M. Zehavi U. Naim M. Polacheck I. Evron R. (1989). Structure-biological activity relationships in alfalfa antimycotic saponins: the relative activity of medicagenic acid and synthetic derivatives thereof against plant pathogenic fungi. *J. Phytopathol.* 125 209–216.

[B22] Lima F. G. Haraguchi M. Pfister J. A. Guimaraes V. Y. Andradel D. D. F. Ribeiro C. S. (2012). Weather and plant age affect the levels of steroidal saponin and *Pithomyces chartarum* spores in *Brachiaria* grass. *Int. J. Poisonous Plant Res.* 2:8.

[B23] Martyniuk S. Biały Z. (2008). Effects of saponins from *Medicago arabica* on in vitro growth of *Cephalosporium gramineum*. *Phytopathol. Pol.* 49 49–55.

[B24] Moses T. Papadopoulou K. K. Osbourn A. (2014). Metabolic and functional diversity of saponins, biosynthetic intermediates and semi-synthetic derivatives. *Crit. Rev. Biochem. Mol. Biol.* 49 439–462. 10.3109/10409238.2014.953628 25286183 PMC4266039

[B25] Nozzolillo C. Arnason J. T. Campos F. Donskov N. Jurzysta M. (1997). Alfalfa leaf saponins and insect resistance. *J. Chem. Ecol.* 23 995–1002.

[B26] Oleszek W. (1996). “Alfalfa saponins: structure, biological activity, and chemotaxonomy,” in *Saponins Used in Food and Agriculture*, eds Waller G. R. Yamasaki K. (New York, NY: Plenum Press). 10.1007/978-1-4613-0413-5_13 8910702

[B27] Oleszek W. Jurzysta M. Ploszynski M. Colquhoun I. J. Price K. R. Fenwick K. R. (1992). Zahnic acid tridesmoside and other dominant saponins from alfalfa (*Medicago sativa* L.) aerial parts. *J. Agric. Food Chem.* 40 191–196.

[B28] Oleszek W. Nowacka J. Gee J. M. Wortley G. M. Johnson I. T. (1994). Effects of some purified alfalfa (*Medicago sativa*) saponins on transmural potential difference in mammalian small intestine. *J. Sci. Food Agric.* 65 35–39.

[B29] Oleszek W. Price K. R. Colquhoun I. J. Jurzysta M. Ploszynski M. Fenwick G. R. (1990). Isolation and identification of alfalfa (*Medicago sativa* L.) root saponins: their activity in relation to a fungal bioassay. *J. Agric. Food Chem.* 38 1810–1817.

[B30] Paape T. Bataillon T. Zhou P. Kono T. J. Y. Briskine R. Young N. D. (2013). Selection, genome-wide fitness effects and evolutionary rates in the model legume *Medicago truncatula*. *Mol. Ecol.* 22 3525–3538. 10.1111/mec.12329 23773281

[B31] Perez A. J. Kowalczyk M. Simonet A. M. Macias F. A. Oleszek W. Stochmal A. (2013). Isolation and structural determination of triterpenoid glycosides from the aerial parts of alsike clover (*Trifolium hybridum* L.). *J. Agric. Food Chem.* 61 2631–2637. 10.1021/jf305541e 23438309

[B32] Pollier J. Morreel K. Geelen D. Goossens A. (2011). Metabolite profiling of triterpene saponins in *Medicago truncatula* hairy roots by liquid chromatography Fourier transform ion cyclotron resonance mass spectrometry. *J. Nat. Prod.* 74 1462–1476. 10.1021/np200218r 21615146

[B33] Ronfort J. Bataillon T. Santoni S. Delalande M. David J. Prosperi J. M. (2006). Microsatellite diversity and broad scale geographic structure in a model legume: building a set of nested core collection for studying naturally occurring variation in *Medicago truncatula*. *BMC Plant Biol.* 6:28. 10.1186/1471-2229-6-28 17166278 PMC1762007

[B34] Saniewska A. Jarecka A. Biały Z. Jurzysta M. (2006). Antifungal activity of saponins originated from *Medicago hybrida* against some ornamental plant pathogens. *Acta Agrobot.* 59 51–58.

[B35] Sen S. Makkar H. P. Becker K. S. (1998). Alfalfa saponins and their implication in animal nutrition. *J. Agric. Food Chem.* 46 131–140. 10554208 10.1021/jf970389i

[B36] Solíz-Guerrero J. B. Rodríguez-García D. Rodríguez-García R. Angulo-Sánchez L. Méndez-Padilla G. (2002). *Quinoa Saponins: Concentration and Composition Analysis*. Alexandria, VA: ASHS Press.

[B37] Suzuki H. Achnine L. Xu R. Matsuda S. P. Dixon R. A. (2002). A genomics approach to the early stages of triterpene saponin biosynthesis in *Medicago truncatula*. *Plant J.* 32 1033–1048. 12492844 10.1046/j.1365-313x.2002.01497.x

[B38] Szakiel A. Pączkowski C. Henry M. (2011). Influence of environmental abiotic factors on the content of saponins in plants. *Phytochem. Rev.* 10 471–491.

[B39] Tava A. Mella M. Avato P. Argentieri M. P. Bialy Z. Jurzysta M. (2005). Triterpenoid glycosides from leaves of *Medicago arborea* L. *J. Agric. Food Chem.* 53 9954–9965. 16366680 10.1021/jf052468x

[B40] Tava A. Pecetti L. (2012). Chemical investigation of saponins from twelve annual Medicago species and their bioassay with the brine shrimp *Artemia salina*. *Nat. Prod. Commun.* 7 837–840. 22908560

[B41] Tava A. Scotti C. Avato P. (2011). Biosynthesis of saponins in the genus Medicago. *Phytochem. Rev.* 10 459–469.

[B42] Tzin V. Snyder J. H. Yang D. S. Huhman D. V. Watson B. S. Allen S. N. (2019). Integrated metabolomics Identifies CYP72A67 and CYP72A68 Oxidases in the biosynthesis of medicago truncatula oleanate sapogenins. *Metabolomics* 15:85. 10.1007/s11306-019-1542-1 31144047

[B43] Voutquenne L. Lavaud C. Massiot G. Men-Olivier L. L. (2002). Structure-activity relationships of haemolytic saponins. *Pharm. Biol.* 40 253–262.

[B44] Zelena E. Dunn W. B. Broadhurst D. Francis-McIntyre S. Carroll K. M. Begley P. (2009). Development of a robust and repeatable UPLC-MS method for the long-term metabolomic study of human serum. *Anal. Chem.* 81 1357–1364. 10.1021/ac8019366 19170513

[B45] Zhu Z. Liang Z. Han R. Wang X. (2009). Impact of fertilization on drought response in the medicinal herb Bupleurum chinense DC.: growth and saikosaponin production. *Ind. Crops Prod.* 29 629–633.

[B46] Zimmer D. E. Pedersen M. W. McGuire C. F. (1967). A bioassay for *Alfalfa Saponins* using the fungus, *Trichoderma viride* pers. ex Fr.1. *Crop Sci.* 7 223–224.

